# Construction of an Artificial Biosynthetic Pathway for the Styrylpyrone Compound 11-Methoxy-Bisnoryangonin Produced in Engineered *Escherichia coli*

**DOI:** 10.3389/fmicb.2021.714335

**Published:** 2021-08-10

**Authors:** Kyung Taek Heo, Byeongsan Lee, Jae-Hyuk Jang, Jung-Oh Ahn, Young-Soo Hong

**Affiliations:** ^1^Anticancer Agent Research Center, Korea Research Institute of Bioscience and Biotechnology, Cheongju-si, South Korea; ^2^Department of Bio-Molecular Science, KRIBB School of Bioscience, University of Science and Technology (UST), Daejeon, South Korea; ^3^Biotechnology Process Engineering Center, KRIBB, Cheongju-si, South Korea

**Keywords:** styrylpyrone, 11-methoxy-bisnoryangonin, styrylpyrone synthase, *de novo* biosynthesis, type III PKS

## Abstract

A cDNA clone (named *pnpks*), which shows high homology to the known chalcone synthase (CHS)-like type III PKS, was obtained from the leaves of *Piper nigrum*. The PnPKS protein with ferulic acid catalyzed lactonization instead of chalcone or stilbene formation. The new product was characterized as a styrylpyrone, 11-methoxy-bisnoryangonin, which is the lactonization compound of a linear triketide formed as the reaction product of PnPKS protein with ferulic acid. These results show that *pnpks* encodes a styrylpyrone synthase (SPS)-like PKS that catalyzes two-chain elongation with feruloyl CoA-linked starter substrates. Although these styrylpyrone compounds are promising for use in human healthcare, they are mainly obtained by extraction from raw plant or mushroom sources. For *de novo* synthesis of 11-methoxy-bisnoryangonin in the heterologous host *Escherichia coli* from a simple sugar as a starter, the artificial biosynthetic pathway contained five genes: *optal*, *sam5*, *com*, and *4cl2nt*, along with the *pnpks* gene. The engineered _L_-tyrosine overproducing *E. coli* ∆COS1 strain, in which five biosynthetic genes were cloned into two vectors, pET-opT5M and pET22-4P, was cultured for 24 h in a minimal glucose medium containing ampicillin and kanamycin. As a result, 11-methoxy-bisnoryangonin production of up to 52.8 mg/L was achieved, which is approximately 8.5-fold higher than that in the parental *E. coli* strain harboring a plasmid for 11-methoxy-bisnoryangonin biosynthesis. As a potential styrylpyrone compound, 11-methoxy-bisnoryangonin, was successfully produced in *E. coli* from a simple glucose medium, and its production titer was also increased using engineered strains. This study provides a useful reference for establishing the biological manufacture of styrylpyrone compounds.

## Introduction

Styrylpyrones are among the most abundant metabolites found in *Piper methysticum*, a crop of the Pacific Islands, suggesting that their underlying biosynthetic mechanism likely emerged after the diversification of the *Piper* genus (Whitton et al., 2003). In many island regions of the Pacific Ocean, the root extract of the plant has traditionally been used as a drink with anesthetic and sedative properties. Recently, there have been reports of styrylpyrone kavain, the major compound of the root extract of *P. methysticum*, which directly interacts with γ-aminobutyric acid type A receptors (Chua et al., 2016; Kautu et al., 2017). Thus, the anxiolytic effect of *P. methysticum* is likely attributable to the pharmacological effects of α-pyrones on the nervous system (Bilia et al., 2002). Various mushrooms have also been reported to produce different styrylpyrones, which have shown important biological effects, such as anti-diabetic, anti-oxidative, anti-platelet, anti-inflammatory, anti-cancer, and antiviral activities (Lee and Yun, 2011). Thus, styrylpyrones hold great promise as natural compounds for food and medical industries to replace synthetic single compounds and extracts of plants that have been implicated in medical conditions. However, plants usually contain complex styrylpyrone mixtures, which are difficult to separate into larger amounts of desired individual compounds. In contrast, the functional combination of plant-specific biosynthetic pathways into microorganisms allows for the production of individual styrylpyrones as a single compound, which can be synthesized in large amounts and isolated more easily (Facchini et al., 2012; Wang et al., 2016; Noda and Kondo, 2017; Palmer and Alper, 2019).

Styrylpyrone synthase (SPS), a chalcone synthase (CHS)-like plant-specific type III polyketide synthase (PKS), normally accepts a 4-coumarate-CoA ligase (4CL)-generated 4-coumaroyl-CoA thioester as a starter substrate and catalyzes the iterative decarboxylative condensation of two-carbon ketone units derived from its extender substrate, malonyl-CoA (Austin and Noel, 2003; Abe and Morita, 2010; Yu et al., 2012). In most cases, the linear triketide intermediate cyclizes *via* Claisen condensation to form the aromatic chalcone backbone. However, SPS yields a triketide intermediate that lactonizes to produce the pyrone backbone (Pluskal et al., 2019). On behalf of typical CHS, functionally different CHS-like PKSs have been found in plants. Their amino acid sequences are highly homologous and likely belong to a family of type III PKSs, which show similarities in catalytic domains. However, studies are still ongoing to identify the amino acid residues that affect the specificity of the initial substrate and/or the final product (Jez et al., 2000; Morita et al., 2011; Stewart et al., 2017).

**Figure 1:**
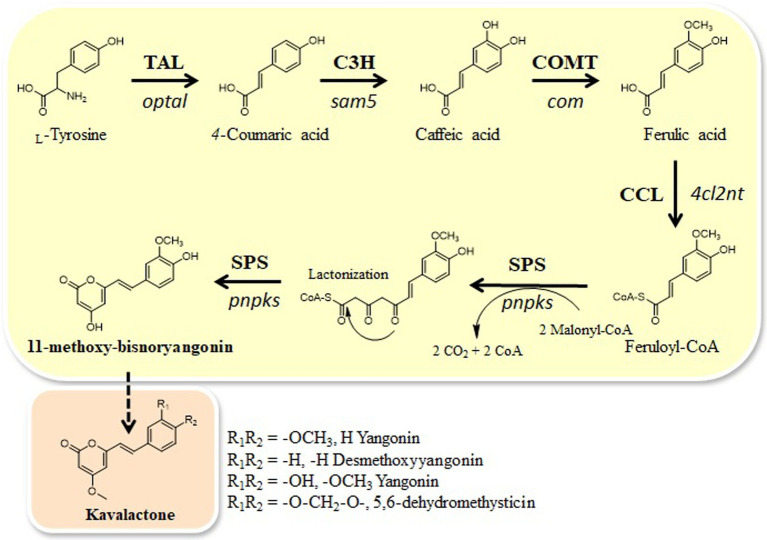
Engineered biosynthetic pathways for styrylpyrone compound, 11-methoxy-bisnoryangonin, starting from _L_-tyrosine in *E. coli* and the proposed kavalactone biosynthetic pathway. Tyrosine ammonia-lyase (TAL) gene (*optal*), C3H (4-coumarate 3-hydroxylase) gene (*sam5*), COMT (caffeic acid *O*-methyltransferase) gene (*com*), CCL gene (*4cl2nt*), and SPS gene (*pnPKS*). Bold arrows indicate the major synthetic pathway used in this study.

Here, we identified one CHS-like gene, *pnpks*, in the black pepper (*Piper nigrum*) transcriptome. We found that *pnpks* encodes a protein with SPS enzymatic activity that condenses two malonyl-CoA with feruloyl CoA-linked starter substrate and further cyclizes to pyrone ring, resulting in the formation of 11-methoxy-bisnoryangonin. In addition, we constructed a microbial *de novo* biosynthetic pathway to produce 11-methoxy-bisnoryangonin using this *pnpks* gene. 11-methoxy-bisnoryangonin was synthesized by five enzymes expressed in plasmids (pET-opT5M and pET22-4P) from intracellular _L_-tyrosine. The pET-opT5M vector, containing the *optal*, *sam5*, and *com* genes, is responsible for the production of ferulic acid (Kang et al., 2015), while the pET22-4P vector, which contains the *4cl2nt* and *pnpks* genes, converts ferulic acid to 11-methoxy-bisnoryangonin (Figure 1). For the efficient production of 11-methoxy-bisnoryangonin in *E. coli*, the engineered _L_-tyrosine overproducing *E. coli* ∆COS1 strain (Kang et al., 2015), which harbors the artificial biosynthetic gene cluster (pET-opT5M and pET22-4P), was cultured for 24 h in a minimal glucose medium containing kanamycin and ampicillin. As a result, 11-methoxy-bisnoryangonin production as high as 52.8 mg/L was achieved after 24 h of culture, this level is approximately 8.5-fold higher than that in the parental *E. coli* strain harboring five genes in two plasmids.

## Materials and Methods

### Bacterial Strains, Plasmids, and Chemicals

The bacterial strains and plasmids used in this study are listed in Table 1. Antibiotics were added to the medium at the following concentrations: kanamycin, 50 mg/L; ampicillin, 100 mg/L. Ferulic acid, cinnamic acid, 4-coumaric acid, and caffeic acid were purchased from Sigma-Aldrich (United States) and used as substrates for *in vitro* and/or *in vivo* reactions and standard for product identification by high-performance liquid chromatography (HPLC). Coenzyme A (CoA), adenosine triphosphate (ATP), and malonyl-CoA were purchased from Sigma-Aldrich for enzyme activity assays. The restriction enzymes (NEB, United States; Takara, Japan), AccuPower Ligation kit (Bioneer, Korea), and KOD-plus-DNA polymerase (TOYOBO, Japan) were used according to the manufacturer’s instructions.

**Table 1:** Plasmids and strains used in this study.

Plasmids	Relevant characteristics	References
pET-28a(+)	pBR322 origin, f1 origin, T7 promoter, Kan^R^	Novagen
pET-22b(+)	pBR322 origin, f1 origin, T7 promoter, Amp^R^	Novagen
pMD19-T	ColE1 origin, *lacZα*, Amp^R^	Takara
pMD-PnPKS	pMD19-T carrying PKS gene (*pnpks*) from *Piper nigrum* cDNA library	This study
pET-PnPKS	pET-28a(+) carrying PKS gene (*pnpks*) from *Piper nigrum*	This study
pET-4CL2nt	pET-22b(+) carrying codon-optimized 4CL gene (*4cl2nt*) from *Nicotiana tabacum*	Kang et al. (2012)
pET-opT5M	pET-28a(+) carrying codon-optimized TAL gene (*optal*), codon-optimized C3H gene (*sam5*) from *Saccharothrix espanaensis*, and COMT gene (*com*) from *Arabidopsis thaliana*	Kang et al. (2015)
pET22-4P	pET-22b(+) carrying *4cl2nt* and *pnpks*	This study
Strains		
*E. coli* DH5α	For cloning	Invitrogen
*E. coli* C41(DE3)	Derivative strain of *E. coli* BL21(DE3)	Miroux and Walker (1996)
∆COS1	Derivative strain of *E. coli* C41(DE3); Δ*tyrR*::*tyrA*^fbr^, *aroG*^fbr^	Kang et al. (2015)
P1	*E. coli* C41(DE3) harboring pET22-4P	This study
P2	*E. coli* C41(DE3) harboring pET-opT5M and pET22-4P	This study
P3	∆COS1 harboring pET-opT5M and pET22-4P	This study
COS6-T5M	*E. coli* C41(DE3); Δ*tyrR*::*tyrA*^fbr^, *aroG*^fbr^; Δ*bioC*::*optal*, *sam5*, *com*	Kang et al. (2018)
P4	COS6-T5M harboring pET22-4P	This study

Amp^R^, ampicillin; Km^R^, kanamycin.

### Isolation of the *pnpks* Gene

The *pnpks* gene was identified through a CHS homology search on the black pepper (*P. nigrum*) fruit transcriptome data as described previously (Jin et al., 2018, 2020). Gene-specific primer pairs were designed from the candidate genes to clone the respective open reading frames (ORFs) from the cDNA library. The deduced amino acid sequence of the *pnpks* gene showed the same amino acid sequences as the CHS (MK058495) originating from *P. methysticum* already deposited in GenBank (Pluskal et al., 2019). PCR products were purified using an IncloneTM Gel & PCR purification kit (Inclone Biotech, Korea) and finally cloned into pMD19-T vector (pMD-pnPKS; Takara, Japan).

### Expression and Purification of His-Tagged PnPKS and 4CL Enzyme

**Figure 2:**
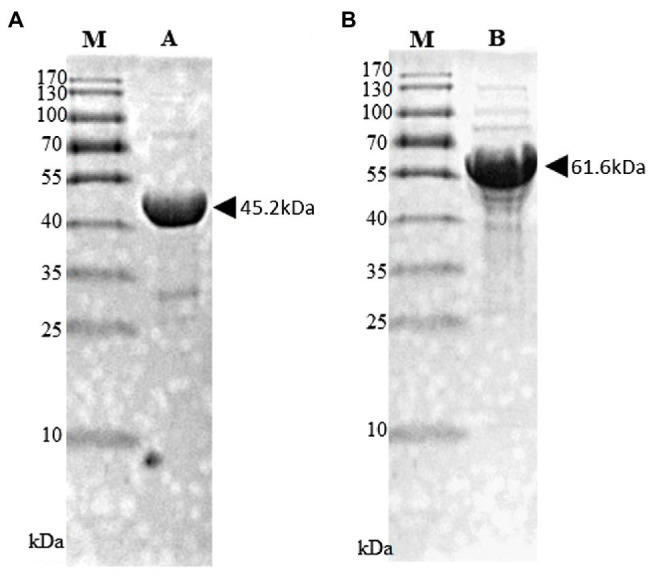
SDS-PAGE analysis of purified His-tagged PnPKS **(A)** and 4CL2nt **(B)**. Purified His-tagged PnPKS (Lane A; 45.2 kDa) and 4CL2nt (Lane B; 61.6 kDa) protein after affinity purification. M, protein size marker.

The black pepper CHS (*pnpks*) gene was cloned into the expression plasmid, pET28a(+), at the *Nde*I and *Hind*III double digestion sites with a 6 × His tag at the N-terminus. The *pnpks* genes were amplified with primers pnPKS_F (5'-CAT**ATG**TCGAAGACGGTAGAGGAGATTC-3', with an *Nde*I site shown by underlining and the start codon of *pnpks* shown in boldface letters) and pnPKS_R (5'-AAGCTTAGTTGGCCTCGGCG-3', with a *Hind*III site shown by underlining) and cloned into pET-28a(+), which resulted in pET-PnPKS. The *4cl2nt* gene (4-coumarate, CoA ligase gene from *Nicotiana tabacum*; GenBank # AAB18638) was cloned into pET-28a(+), resulting in pET-4Cl2nt, as described previously (Kang et al., 2012). *E. coli* C41 (DE3) cells transformed with pET-PnPKS or pET-4Cl2nt were inoculated in 100 ml of *Luria-Bertani* (LB) medium supplemented with kanamycin and cultured at 37°C. When the optical density of the culture broth at 600 nm (OD_600_) was 0.6, isopropyl-D-thiogalactopyranoside (IPTG, 1 mM) was added to induce protein expression. After culturing for 16 h at 26°C, the cells were harvested by centrifugation (2,000 × *g*, 20 min, 4°C). The cells were resuspended in 4 ml of lysis buffer [50 mM Tris-HCl (pH 7.4), imidazole 10 mM, and NaCl 100 mM] with a protease inhibitor cocktail (Sigma-Aldrich, United States). The harvested cells were centrifuged at 15,000 × *g* for 10 min at 4°C after sonic treatment. The supernatant was mixed with His-Hyper agarose resin (Lugen Sci. Co., Korea) in a Poly-Prep chromatography column (Bio-Rad, United States) for 1 h. The mixed resins were washed with 50 ml of wash buffer containing 50 mM imidazole, and the His-tagged enzymes were eluted using 2 ml of an elution buffer containing 250 mM imidazole. The purified His-tagged enzymes were dialyzed against 10 mM Tris-HCl (pH 8.0) containing 0.1 mM EDTA, 0.1 mM DTT, and 10% glycerol. The purified enzyme concentrations were estimated by the Bradford method, using the Coomassie Plus Protein Assay kit (Bio-Rad, United States). The purified His-tagged PnPKS (45.2 kDa) and 4CL2nt (61.6 kDa) proteins were analyzed using 12% (w/v) sodium dodecyl sulfate (SDS)-polyacrylamide gel electrophoresis (PAGE) (Figure 2).

### Enzymatic Assay of PnPKS With Phenolic Acids

Reaction mixtures (100 μl) containing Tris-HCl (50 mM, pH 8.0), CoA (0.1 mM), malonyl-CoA (0.2 mM), ATP (5 mM), MgCl_2_ (1 mM), and all phenolic acids (cinnamic acid, 4-coumaric acid, caffeic acid, and ferulic acid; 0.1 mM) with both His-tagged 4CL2nt (1 μM) and PnPKS (1 μM) were incubated at 30°C for 4 h. The reaction mixture was extracted with an equal volume of ethyl acetate. The organic layer was then evaporated to dryness. The residual material was dissolved in 100 μl of methanol. Twenty microliters of the extract was applied to an HPLC and liquid chromatography/mass spectrometry (LC/MS) system, as described below.

### Construction of 11-Methoxy-Bisnoryangonin Expression Vector

Using pET-PnPKS as a template, the *pnpks* gene was amplified by PCR using the primers Nspe (5'-ACTAGTAGGTTGAGGCCGTTGAGCACCGCC-3', which is located upstream of the T7 promoter region of the pET-28a(+) vector and contains the designed *Spe*I site shown by underlining) and pnPKS_R (5'-AAGCTTAGTTGGCCTCGGCG-3', with a *Hind*III site shown by underlining). Using pET-4CL2nt as a template, the *4cl2nt* gene was amplified with primers 4cl2nt_F (5'-CAT**ATG**GAGAAAGACACGAAGCAAGTTGACATC-3', with an *Nde*I site shown by underlining and the start codon of *4cl2nt* shown in boldface letters) and Cspe (5'-ACTAGTTCCTCCTTTCAGCAAAAAACCCCTC-3', the sequence is located downstream of the terminator region of the pET-28a(+) vector and contains the *Spe*I site shown by underlining). Each of the amplified fragments was digested with corresponding sites and cloned between the *NdeI- and Hind*III-digested pET-22b(+), resulting in pET22-4P. The pET-opT5M vector for ferulic acid production has been previously reported (Kang et al., 2012).

### Culture Conditions for 11-Methoxy-Bisnoryangonin Production and Bioconversion

Engineered *E. coli* strains were grown at 37°C in LB medium containing appropriate antibiotics (100 μg/ml ampicillin for pET22-4P and 50 μg/ml kanamycin for pET-opT5M). The overnight culture was inoculated into a fresh LB medium supplemented with kanamycin and ampicillin. The culture was grown at 37°C to an OD_600_ of 0.6, and IPTG was added to a final concentration of 1 mM, followed by incubation at 26°C for 6 h. The cultured cells were harvested and resuspended in 30 ml minimal glucose medium (SM; 7.3 g/L K_2_HPO_4_, 3 g/L KH_2_PO_4_, 8.4 g/L MOPS, 0.5 g/L NaCl, 2 g/L NH_4_Cl, 5 g/L MgSO_4_ 7H_2_O, 5 g/L (NH_4_)_2_SO_4_, 0.1 ml/L trace elements, 15 g/L glucose, 1 mM IPTG, and appropriate antibiotics) in 250-ml flasks. The resuspended cells were incubated at 26°C for 48 h. For the bioconversion experiments, the recombinant *E. coli* strains that harbored the pET22-4P plasmid were cultured in the SM media supplemented with 0.1 mM each of cinnamic acid, 4-coumaric acid, caffeic acid, and ferulic acid. The samples were collected after 24 h. The cultured samples were collected over time and analyzed by HPLC and LC/MS.

### Isolation of 11-Methoxy-Bisnoryangonin

The recombinant *E. coli* strains harboring the pET22-4P plasmid were cultured using the same method as described earlier, and the culture volume and time were increased to 2 L for 60 h. After ferulic acid (15 mg/L) supplementation, the ethyl acetate soluble material was further separated using semi-preparative HPLC (Waters Atlantis T3 C18 column: 10 × 250 mm, 5 μm) with a gradient solvent system (35% CH_3_CN–H_2_O [0.05% trifluoroacetic acid (TFA)] to 45% CH_3_CN–H_2_O (0.05% TFA) over 15 min, UV 254 nm detection, flow rate: 3 ml/min). Finally, pure 11-methoxy-bisnoryangonin (5.2 mg) was eluted at 13.2 min. The structure of 11-methoxy-bisnoryangonin was determined based on ^1^H and ^13^C NMR data with values reported in the literature (Supplementary Table S1; Zan et al., 2011).

### Quantification of 11-Methoxy-Bisnoryangonin

The cultures were extracted using an equal volume of ethyl acetate. Ethyl acetate extracts were then dried and resuspended in methanol. Twenty microliters of the extract was injected into a J’sphere ODS-H80 column (4.6 × 150 mm i.d., 5 μm; YMC, Japan) using an UltiMate U3000 system (CH_3_CN–H_2_O (0.05% TFA) 20–100% acetonitrile (CH_3_CN) for 20 min and 100% CH_3_CN for 5 min, at a flow rate of 1 ml/min; Thermo Fisher Scientific, Unites States) equipped with a photodiode array detector. The quantification of the compounds was based on the peak areas of absorbance at 320 nm. The quantification data shown in this study were generated from independent experiments performed in triplicate. For LC/MS analysis, samples were dissolved in methanol and analyzed by electrospray ionization (ESI) using an UltiMate U3000-LTQ XL linear ion trap mass spectrometer system (Thermo Fisher Scientific, United States). A 2-μl volume of the sample was applied to an ACQUITY UPLC HSS T3 C18 column (2.1 × 150 mm; 2.5 μm particle size; Waters, United States), and the mobile phase used for the linear gradient condition (5–100% CH_3_CN–H_2_O containing 0.1% formic acid for 15 min, 100% CH_3_CN for 5 min, at a flow rate 0.3 ml/min). The mass spectrometry experiments were controlled and analyzed using menu-driven software provided with the Xcalibur system (version 2.2 SP1.48; Thermo Fisher Scientific).

## Results

### Identification of CHS-Like Gene (*pnpks*) From *Piper nigrum*

**Figure 3:**
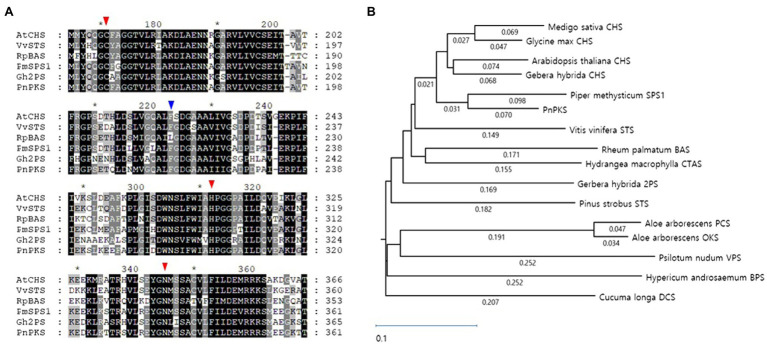
Alignment **(A)** and phylogenetic analysis **(B)** of PnPKS against known CHS-superfamily type III PKS. **(A)** The red triangle indicates the three amino acids forming a Cys-His-Asn catalytic triad that is essential for starter substrate loading and malonyl-CoA condensation. The blue triangle indicates a “gate-keeper” that is known to adjust the chain elongation. **(B)** Arrow indicates PnPKS in this study. The sources and GenBank accession numbers are AtCHS from *Arabidopsis thaliana* (P13114), VvSTS from *Vitis vinifera* (ABE68894), RpBAS from *Rheum palmatum* (AAK82824), PmSPS1 from *Piper methysticum* (QCX36371), Gh2PS from *Gerbera hybrida* (P48391), CHS from *Medigo sativa* (AAB41559), *Glycine max* (P19168.1), and *Gerbera hybrid* (P48390.1), CTAS from *Hydrangea macrophylla* (BAA32733.1), OKS from *Aloe arborescens* (Q3L7F5.1), PCS from *Aloe araborescens* (Q58V97.1), VPS from *Psilotum nudum* (Q9SLX9.1), STS from *Pinus strobus* (P480407.1), BPS from *Hypericum androsaemum* (Q8SAS8.1), and DCS from *Curcuma longa* (C0SVZ5.1). The nucleotide sequence of the *pnpks* gene from *Piper nigrum* is provided in the Supplementary data section. Amino acid sequences were aligned using the ClustalW method and the phylogenetic tree constructed by using the MegAlign program of the DNASTAR Lasergene software (DNASTAR Inc.).

*Piper nigrum* (black pepper) produces an alkaloid compound, piperine, which is derived from the polyketide pathway (Geisler and Gross, 1990; Srinivasan, 2007). The corresponding PKS that provides the piperine polyketide chain has not been identified, but it is estimated that the CHS-like type III PKS can produce it (Jin et al., 2020; Schnabel et al., 2020). In our investigation of piperine biosynthesis, we identified one CHS-like gene, *pnpks*, in the *P. nigrum* transcriptome data. The deduced length of the enzymes encoded by the *pnpks* gene was 389 amino acids. Phylogenetic analyses revealed that PnPKS was clustered together with other plant CHS-superfamily type III PKSs (Figure 3). The PnPKS was closer to that of typical type III PKSs [CHS and STS (stilbene synthase)] from *Arabidopsis thaliana* and *Vitis vinifera*, with identities of 79.7 and 73.5%, respectively. In addition, the deduced amino acid sequence showed more than 66% identity with those of other type III PKSs of plant origin: 81.5% identity with *P. methysticum* SPS, 68.4% identity with *Gerbera hybrida* 2-pyrone synthase (2-PS), and 66.1% identity with *Rheum palmatum* benzalacetone synthase (BAS). PnPKS maintains a CoA-binding site similar to that of the catalytic triad of Cys169, His309, and Asn342 [AtCHS (CHS from *A. thaliana*) numbering; Ferrer et al., 1999; Figure 3]. The “gate-keeper” Phe220 is also located at the junction between the CoA-binding tunnel and the active-site cavity. The presence of these conserved functional motifs is thought to facilitate repeated decarboxylation reactions and help orient substrates and intermediates during the condensation reactions. However, other members of the CHS family with very similar amino acid sequences, such as SPS, 2-PS, STS, and BAS, result in the formation of different polyketides using various starting substrates, condensation reactions, and cycling processes. It is of interest to understand how these similar but distinct enzymes function to produce specific final products. To date, very little is known about the amino acid residues responsible for their mode of cyclization and starter specificity. Therefore, it is difficult to determine the end product of the newly acquired PnPKS using its amino acid sequence alone.

### Characterization of the SPS Function With Ferulic Acid of the PnPKS

**Figure 4:**
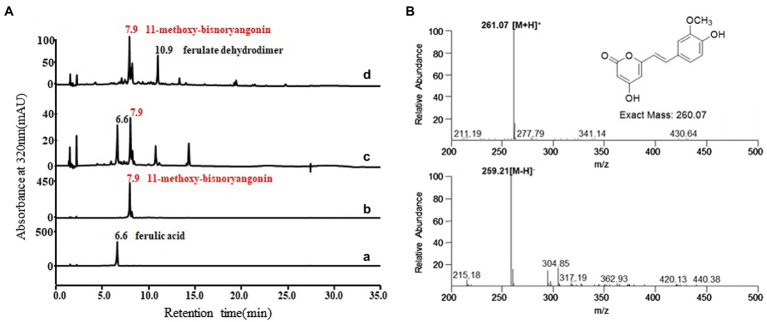
Functional analysis of PnPKS using *in vitro* enzymatic reaction and bioconversion experiment with ferulic acid. **(A)** HPLC profile of the standard ferulic acid (a), purified 11-methoxy-bisnoryangonin (b), *in vitro* reaction (c), and ferulic acid supplemented *E. coli* harboring pET22-4P (P1; d). The absorbance was monitored at 320 nm. **(B)** Selected mass ion chromatogram of peak at 7.9 min (trace c) exhibited *m*/*z* 261 [M+H]^+^ and *m*/*z* 259 [M−H]^−^,which corresponded to 11-methoxy-bisnoryangonin. Peak at 10.9 min (trace d) exhibited *m/z* 371 [M+H]^+^, which corresponded to dimerized ferulic acid.

To identify the function of the *pnpks* gene originating from *P. nigrum*, we assessed the enzymatic activity using purified His-tagged PnPKS with 4-coumarate-CoA ligase (4CL, Figure 2). Based on the previous literature, the 4CL from *Nicotiana tabacum* (4CL2nt) has already been identified as having a broad substrate specificity for cinnamic acid, 4-coumaric acid, caffeic acid, and ferulic acid. In addition, the relative specificities of 4CL2 toward cinnamic acid, caffeic acid, and ferulic acid are 29, 25, and 62%, respectively, compared to the value of 4-coumaric acid (Lee and Douglas, 1996). The reaction of ferulic acid with the 4CL2nt enzyme in the presence of PnPKS led to the formation of a clear new peak at 7.9 min, which was detected using HPLC [Figure 4A(c)]. The molecular weight of the new peak was determined using LC/MS. The peak at 7.9 min exhibited parent mass ion peaks at *m/z* 261 [M+H]^+^ and *m/z* 259 [M–H]^−^ (Figure 4B), which corresponded to a molecular weight of 260 Da, and significant amounts of ferulic acid that was used as substrate were also detected at 6.6 min. The PnPKS protein with ferulic acid did not catalyze the formation of chalcone (proposed MW 302 Da) or stilbene (proposed MW 258 Da) compounds. In addition, we constructed a bioconversion system utilizing the *pnpks* gene and the *4cl2nt* gene. The *pnpks* and *4cl2nt* genes were cloned into the expression vector pET-22b using a previously described method, resulting in pET22-4P (Table 1). Ferulic acid was added to the cultured recombinant *E. coli* C41 (DE3) strain (P1) harboring *pnpks* and *4cl2nt* genes (pET22-4P). The P1 culture broth and bacterial cells were collected after 24 h of culture and were subjected to HPLC analysis [Figure 4A(d)]. As a result of the enzymatic reaction, the 7.9 min peak was also identified and the MW was the same at 260 Da.

**Figure 5:**
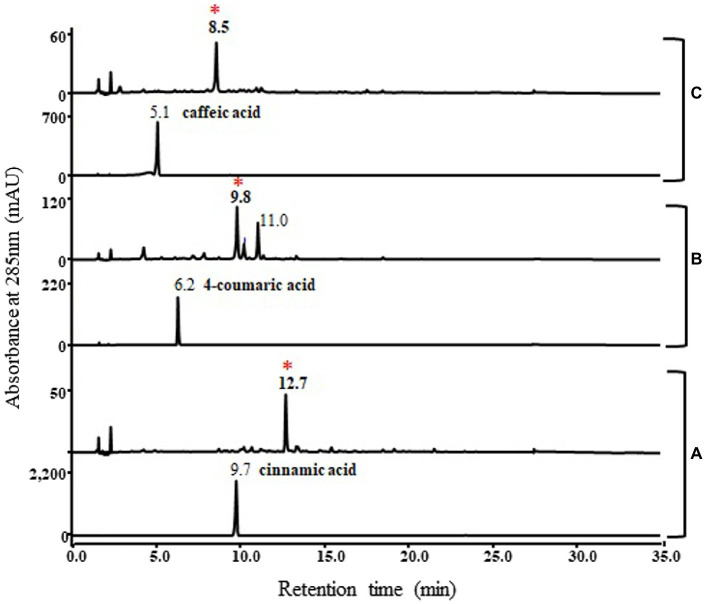
HPLC profile of bioconversion experiments with cinnamic acid **(A)**, 4-coumaric acid **(B)**, and caffeic acid **(C)**. Lower panels represent standard phenylpropanoic acids and upper panels represent reaction results. The absorbance was monitored at 280 nm. Cinnamic acid, 4-coumaric acid, and caffeic acid formed new peaks (12.7 min, 9.8 min, and 8.5 min, respectively) corresponding to the molecular weights of different chalcone compounds, which are denoted by asterisks. The mass spectra of the asterisked peaks are shown in Supplementary Figure S1.

In addition, the functional study of this pnPKS enzyme against other phenylpropanoic acids, cinnamic acid, 4-coumaric acid, and caffeic acid was also conducted under the same bioconversion conditions as those used for ferulic acid (Figure 5). As a result, cinnamic acid, 4-coumaric acid, and caffeic acid formed new peaks (at RT 12.7 min, 9.8 min, and 8.5 min, respectively) corresponding to the molecular weights of chalcone compounds (Supplementary Figure S1), which are denoted by asterisks on Figure 5. In the case of cinnamic acid and caffeic acid, the bioconversion clearly produced chalcone compounds (m/z 257 [M+H]+ and m/z 289 [M+H]+, respectively), but the 4-coumaric acid converted to chalcone compound at RT 9.8 min (m/z 273 [M+H]+) and the other peak at RT 11.0 min (m/z 310 [M+H]+) which probable corresponded to dehydrodimer (Supplementary Figure S1). Although chalcone compounds were major products in bioconversion experiment using cinnamic acid, 4-coumaric acid, and caffeic acid, but *in vitro* enzyme reactions produced unknown peaks along with the different chalcone compounds (denoted by asterisk), respectively (Supplementary Figure S2). Interestingly, only ferulic acid does not produce chalcone compound as major peak in these reactions.

To obtain the required amount for structural analysis of the 260 Da product estimated to be a pyrone ring, 30 mg of ferulic acid was added to a 2 L fermentation broth of the P1 strain. The structure of the purified 7.9 min peak was identified as 11-methoxy-bisnoryangonin through spectral data interpretation and compared with the values reported in the literature (Supplementary Table S1; Zan et al., 2011). In addition, the other peak at 10.9 min exhibited *m/z* 371 [M+H]^+^, which corresponded to ferulate dehydrodimer [Figure 4A(d)]. The results of the tandem MS analysis suggested that the compound formed an ester bond between the carboxyl residue of one ferulic acid molecule and the hydroxyl group located in the aromatic ring of another ferulic acid (Supplementary Figure S3). Generally, dimerization of ferulic acid provides a pathway for cross-linking polysaccharide chains in some plants (Ralph et al., 1994). A reactive radical can abstract a hydrogen atom from the carboxyl group of ferulic acid to form a phenoxy radical that is highly resonance-stabilized. It is estimated that oxidative coupling *via* the action of peroxidases in *E. coli* cells produces dehydrodimers. These dimer compounds have often been identified in bioconversion experiments with phenylpropanoic acids and are thought to be shunt products.

Taken together, the newly formed 7.9 min peak in *in vitro* enzymatic reaction and bioconversion experiment was identified as 11-methoxy-bisnoryangonin. Thus, the PnPKS protein has SPS enzymatic activity that catalyzes the sequential condensation of one feruloyl-CoA and two malonyl-CoA molecules to form a triketide intermediate, followed by ring closure of the triketide to form a styrylpyrone (Figure 1).

### Construction of *de novo* Artificial Biosynthetic Pathways for 11-Methoxy-Bisnoryangonin

Although styrylpyrone compounds are promising for use in human healthcare, they are mainly obtained by extraction from plants, such as *P. methysticum* or some mushrooms. Therefore, these compounds are important targets for production through the use of engineered microbial strains. Although the aforementioned strains are capable of producing significant levels of 11-methoxy-bisnoryangonin, they require the exogenous addition of the pathway precursor ferulic acid. We attempted a *de novo* synthesis to produce an 11-methoxy-bisnoryangonin compound in *E. coli* by engineering an artificial biosynthetic pathway using the newly identified PnPKS. To this end, three enzyme activities were needed from tyrosine, an essential aromatic amino acid, to engineer the production of ferulic acid in *E. coli*. First, we used a ferulic acid biosynthetic gene cluster (pET-opT5M), consisting of the tyrosine ammonia-lyase (TAL; *optal*) gene, 4-coumarate 3-hydroxylase (C3H; *sam5*) gene, and caffeic acid *O*-methyltransferase gene (COMT; *com*; Table 1; Kang et al., 2012, 2015). The *pnpks* and *4cl2nt* genes (pET22-4P) were used as the next step to produce 11-methoxy-bisnoryangonin from ferulic acid. Each biosynthetic gene was configured to be regulated by the T7 promoter.

**Figure 6:**
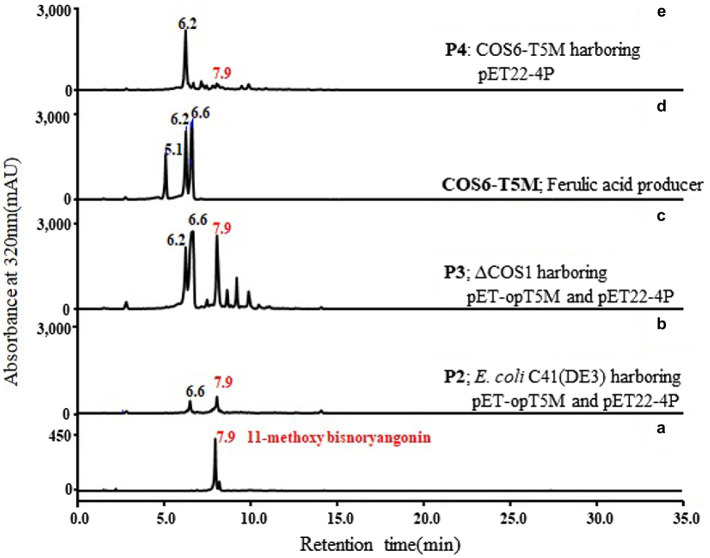
*De novo* biosynthesis of 11-methoxy-bisnoryangonin by the P2, P3, and P4 strains. Comparison of HPLC profiles of purified 11-methoxy-bisnoryangonin (a); the culture broth of P2 (*E. coli* C41 strain harboring pET-opT5M and pET22-4P), (b); P3 (∆COS1 strain harboring pET-opT5M and pET22-4P), (c); COS6-T5M (d); and P4 (COS6-T5M strain harboring pET22-4P), (e). The peaks correspond to caffeic acid (5.1 min), 4-coumaric acid (6.2 min), ferulic acid (6.6 min), and 11-methoxy-bisnoryangonin (7.9 min).

**Figure 7:**
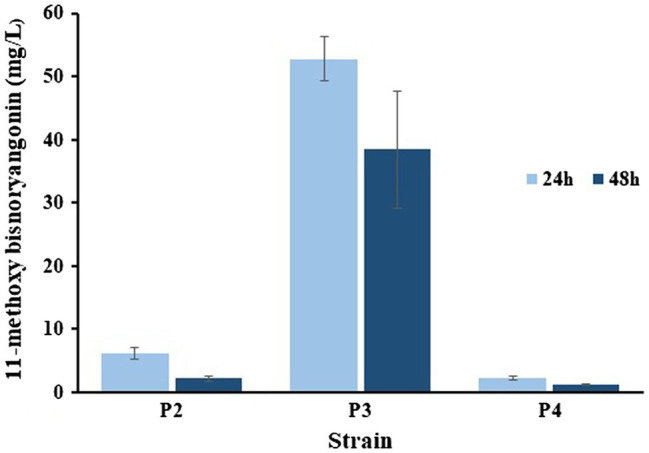
Comparisons of the 11-methoxy-bisnoryangonin productivity. The data were obtained after 24–48 h culture of P2, P3, and P4 strains in a minimal glucose medium. Error bars show one standard deviation from triplicate experiments.

Two plasmids, pET-opT5M and pET22-4P, were simultaneously transformed into *E. coli* C41 (DE3) (P2) and the engineered _L_-tyrosine overproducing strain (P3). The _L_-tyrosine overproducing *E. coli* strain (∆COS1) was engineered to overexpress the chorismate mutase-prephenate dehydrogenase gene (*tyrA*) and D-erythrose-4-phosphate-lyase gene (*aroG*) in a Δ*tyrR* strain background (Kang et al., 2015). A detailed description of the method is provided in the Supporting Information (Supplementary Figure S4). The recombinant strains (P2 and P3) that harbored the artificial biosynthetic gene clusters (pET-opT5M and pET22-4P) were cultured in minimal glucose medium (SM) containing ampicillin and kanamycin in shake flasks at 26°C. The 11-methoxy-bisnoryangonin peaks were detected in the culture broth of the P2 and P3 strains by HPLC analysis (Figure 6). The amount of 11-methoxy-bisnoryangonin in P2 and P3 strain reached 6.2 ± 1.0 mg/L (23.8 μM) and 52.8 ± 3.5 mg/L (203.1 μM) at 24 h (Figure 7), respectively. In addition, the slightly reduced productivity of 11-methoxy-bisnoryangonin in 48 h (Figure 7) was assumed to be due to the instability of the compound. The instability may be attributed to the hydrolysis of esters due to pH changes in the fermentation process. This result means that a *de novo* artificial 11-methoxy-bisnoryangonin biosynthetic pathway was engineered in *E. coli* containing the two-vector system, although previous bioconversion methods include precursor feeding.

In addition, to facilitate cell growth and fermentation in the production of 11-methoxy-bisnoryangonin, we used an engineered *E. coli* strain (COS6-T5M), in which one copy of the ferulic acid production module was inserted into the chromosome of the _L_-tyrosine overproducing strain (∆COS1) (Kang et al., 2018). Using the RedET recombination system, the ferulic acid production module containing the *optal*, *sam5*, and *com* genes was inserted into the *bioC* gene region of the *E. coli* ∆COS1 chromosome, which encodes a malonyl-acyl carrier protein methyltransferase (Supplementary Figure S4). The elimination of this *bioC* gene inhibits the consumption of malonyl-CoA for fatty acid biosynthesis (Lin and Cronan, 2012). The COS6-T5M strain produced ferulic acid under the same culture conditions without the antibiotics [Figure 6(d)]. A total of three peaks, including a peak with the same retention time (6.6 min) as the ferulic acid standard, were detected. The other two peaks were identified as caffeic acid (5.1 min) and 4-coumaric acid (6.2 min). These products can be produced by the reactions of the three enzymes in the artificial ferulic acid pathway. Plasmid pET22-4P was transformed into ferulic acid-producing *E. coli* COS6-T5M (P4) and cultured for 48 h under the same flask culture conditions. However, the strain P4 produced only a small amount of 11-methoxy-bisnoryangonin (2.3 ± 0.2 mg/L at 24 h). This amount is less than that produced by the parental P2 strain (6.2 ± 1.0 mg/L). However, the accumulation of 4-coumaric acid, not ferulic acid, was also identified in the P4 strain [Figure 6(e)], while 4-coumaric acid, caffeic acid, and ferulic acid were produced in the COS6-T5M strain [Figure 6(d)]. Taken together, the final levels of 11-methoxy-bisnoryangonin produced under two-vector conditions from an engineered tyrosine-producing strain (P3) were determined to be 8.5-fold and 22.9-fold over the parental *E. coli* P2 strain and the engineered *E. coli* P4 strain, respectively. The titers of the 11-methoxy-bisnoryangonin of the P2 strain reached 52.8 mg/L after 24 h in flask culture.

## Discussion

In this study, we successfully demonstrated the *de novo* synthesis of a styrylpyrone compound, 11-methoxy-bisnoryangonin, which is an abundant metabolite responsible for the anxiolytic effects of *P. methysticum* extracts. The system was developed using the PnPKS protein as an SPS that catalyzes pyrone formation with specificity to feruloyl-CoA. PnPKS catalyzes the formation of 11-methoxy-bisnoryangonin from malonyl-CoA and ferulic acid precursors when combined with 4CL enzyme. Furthermore, we designed an artificial biosynthetic pathway using the newly identified *pnpks* gene with the other four genes, including those involved in feruloyl-CoA production, to complete the *de novo* synthesis of 11-methoxy-bisnoryangonin in *E. coli*. The production of 11-methoxy-bisnoryangonin from an engineered tyrosine overproducing strain (P3) was 8.5-fold higher than that of the parental *E. coli* (P2) harboring five genes in two plasmids. The titers of 11-methoxy-bisnoryangonin reached 52.8 mg/L in flask culture in a minimal glucose medium. However, strain P4, whose plasmid pET22-4P was transformed into ferulic acid-producing *E. coli* COS6-T5M, produced a smaller amount of 11-methoxy-bisnoryangonin than the parental *E. coli* C41 (DE) harboring five genes in two plasmids (Figure 6). It can be seen that one copy of the modular genes for ferulic acid production shows significantly lower amounts of 11-methoxy-bisnoryangonin when compared to high copy numbers. In general, the plasmid copy number is known to affect the ability of cells to grow and influence cellular metabolism (Karim et al., 2013). Although it is difficult to explain this phenomenon clearly, it can be estimated that an appropriate proportion of enzyme concentrations is required in the heterologous host cell for the optimal rate of biosynthetic metabolic flux (Farasat et al., 2014; Kang et al., 2018). Therefore, this artificial biosynthetic pathway of 11-methoxy-bisnoryangonin is a multi-enzyme reaction that requires a balanced expression of several enzymes. In the future, it will be necessary to improve the expression of these enzymes or culture conditions.

Styrylpyrones hold great promise as natural compounds for food and medical industries to replace synthetic compounds and plant extracts (Lee and Yun, 2011). As a potential styrylpyrone, 11-methoxy-bisnoryangonin was successfully produced in *E. coli* from a glucose medium, and its production titer was also increased using a strain that had an engineered metabolic pathway for tyrosine. The production of plant-originated styrylpyrone compounds, such as 11-methoxy-bisnoryangonin by engineering microbes, suggests the potential for industrialization through further exploration of advanced synthetic biology and metabolic engineering techniques.

## Data Availability Statement

The datasets presented in this study can be found in online repositories. The names of the repository/repositories and accession number(s) can be found in the article/Supplementary Material.

## Author Contributions

KH, BL, J-HJ, and J-OA performed the experimental work, whereas KH, BL, and Y-SH drafted the manuscript. All authors were involved in designing, discussing, and interpreting the results of the experiments.

## Conflict of Interest

The authors declare that the research was conducted in the absence of any commercial or financial relationships that could be construed as a potential conflict of interest.

## Publisher’s Note

All claims expressed in this article are solely those of the authors and do not necessarily represent those of their affiliated organizations, or those of the publisher, the editors and the reviewers. Any product that may be evaluated in this article, or claim that may be made by its manufacturer, is not guaranteed or endorsed by the publisher.
