# Supplementary material for: Construction of an Artificial Biosynthetic Pathway for the Styrylpyrone Compound 11-Methoxy-Bisnoryangonin Produced in Engineered Escherichia coli
Source: Front Microbiol. 2021 Aug 10;12:714335. doi: 10.3389/fmicb.2021.714335 (PMC8388576; doi:10.3389/fmicb.2021.714335)
Supplement: Supplementary file 1 [file Data_Sheet_1.pdf]

SUPPLEMENTAL MATERIAL

Construction of an artificial biosynthetic pathway for the styrylpyrone compound 11-methoxy-bisnoryangonin, produced in engineered *Escherichia coli*

Kyung Taek Heo [1,2], Byeongsan Lee[1], Jae-Hyuk Jang[1,2], Jung-Oh Ahn[3], Young-Soo Hong [1,2,*]

[1]Anticancer Agent Research Center, Korea Research Institute of Bioscience and Biotechnology, 30 Yeongudanji-ro, Ochang-eup, CheongJu-si, Chungbuk 28116, Republic of Korea

[2]Department of Bio-Molecular Science, KRIBB School of Bioscience, University of Science and Technology(UST), Daejeon, Republic of Korea

[3]Biotechnology Process Engineering Center, KRIBB, 30 Yeongudanji-ro, Ochang-eup, CheongJu-si, Chungbuk 28116, Republic of Korea

*** Correspondence:**
Young-Soo Hong
hongsoo@kribb.re.kr

Table S1. ^{13}C (175 MHz) and ^{1}H (700 MHz) NMR spectroscopic data for 11-methoxy-bisnoryangonin.

Figure S1. Selected mass ion chromatogram and the proposed structures of each chalcone peak (denoted by an asterisk) from analyses of reaction products shown in Figure 5. A) Peak at 12.7 min (trace (a) in Figure 5) exhibited *m/z* 257 $[M+H]^+$ and *m/z* 255 $[M-H]^-$, which corresponded to a chalcone compound (MW 256). B) Peak at 9.8 min (trace (b) in Figure 5) exhibited *m/z* 273 $[M+H]^+$ and *m/z* 271 $[M-H]^-$, which corresponded to a chalcone compound (MW 272). C) Peak at 8.5 min (trace (c) in Figure 5) exhibited *m/z* 289 $[M+H]^+$ and *m/z* 287 $[M-H]^-$, which corresponded to a chalcone compound (MW 288). D) Peak at 11.0 min (trace (b) in Figure 5) exhibited *m/z* 311 $[M+H]^+$ and *m/z* 309 $[M-H]^-$, which corresponded to 4-coumarate dehydrodimer (MW 310).

Figure S2. HPLC profile and the proposed structures of *in vitro* enzymatic reactions with cinnamic acid (A), 4-coumaric acid (B), and caffeic acid (C). Lower panels represent standard phenylpropanoic acids and upper panels represent reaction results. The absorbance was monitored at 280 nm. Cinnamic acid, 4-coumaric acid, and caffeic acid formed new peaks (12.7 min, 9.8 min, and 8.5 min, respectively) corresponding to the molecular weights of different chalcone compounds, which are denoted by asterisks.

Figure S3. LC/MS/MS analysis of dimer compound. (A) The peak at 10.9 min exhibited parent mass ion peaks at *m/z* 371 $[M + H]^+$ and *m/z* 369 $[M - H]^-$, which corresponded to molecular weight 370 Da. (B) Structure and MS/MS spectra of the predicted dimer compound.

Figure S4. Schematic illustration showing the strategies for constructing the genome engineered strain.

A) Construction of the L-tyrosine overproducing strain of *E. coli* (ΔCOS1) was achieved by extra gene insertion of *aroG* and *tyrA*, feedback-inhibition resistance (fbr) genes on the *tyrR* gene locus in C41(DE3) strain (Kang *et al.* 2015). B) Construction of the ferulic acid overproducing strain of *E. coli* (COS6-T5M) was achieved by extra gene insertion of T5M module (*optal*, *sam5*, and *com* gene) on the *bioC* gene locus in ΔCOS1 strain (Kang *et al.* 2018).

Table S1 . *13 C (175 MHz) and 1H (700 MHz) NMR Spectroscopic Data for 11-methoxyl-bisnoryangonin..*

	hispdin[a]		11-methoxyl-bisnoryangonin[b]	
Position	δ_c	δ_H (J in Hz)	δ_c	δ_H (J in Hz)
2	168.82		170.4	
3	89.50	5.23(1H, s)	89.3	5.30(1H, d, 2.0)
4	169.85		159.9	
5	101.30	6.13(1H, s)	100.5	6.13(1H, d, 2.0)
6	160.35		163.1	
7	116.91	6.67(1H, d, 16)	116.7	6.84(1H, d, 16.0)
8	134.81	7.12(1H, d, 16)	134.7	7.22(1H, d, 16.0)
9	127.26		126.8	
10	114.47	7.03(1H, d, 2.0)	110.7	7.27(1H1 d, 1.9)
11	145.95		148.4	
12	147.88		148.0	
13	116.16	6.77(1H, d, 8.1)	115.7	6.79(1H, d, 8.1)
14	120.73	6.95(1H, dd, 8.1;2.0)	122.0	7.07(1H, dd, 8.2,;1.8)
10-OCH3			55.7	3.82(3H, s)

[a]Li-feng ZAN et al. (2011) for the literature values of ^{1}H and ^{13}C NMR data

[b]11-methoxyl-bisnoryangonin in DMSO-d_6 (700MHz for ^{1}H and 175MHz for ^{13}C NMR data)

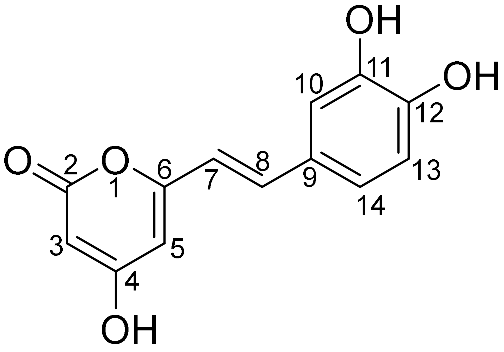

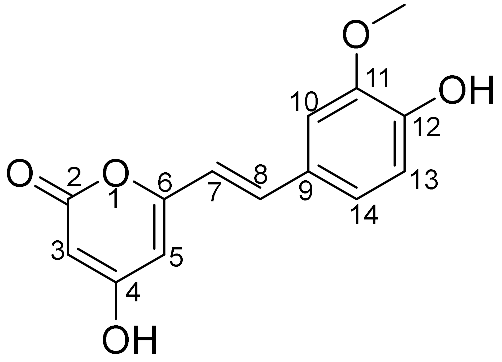

Figure S1. Selected mass ion chromatogram and the proposed structures of each chalcone peak (denoted by an asterisk) from analyses of reaction products shown in Figure 5.

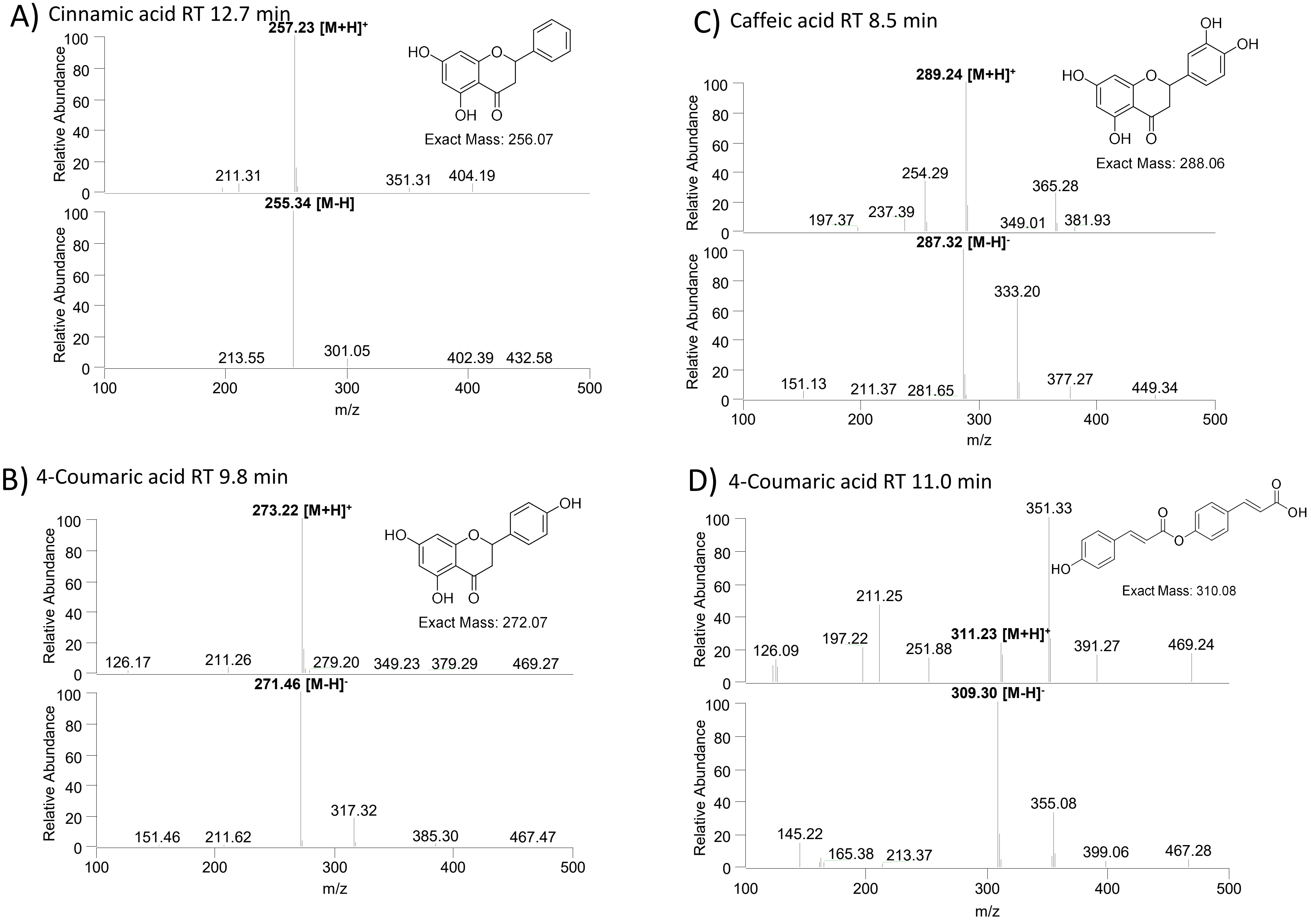

Figure S2. HPLC profile of *in vitro* enzymatic reactions with cinnamic acid (A), 4-coumaric acid (B), and caffeic acid (C).

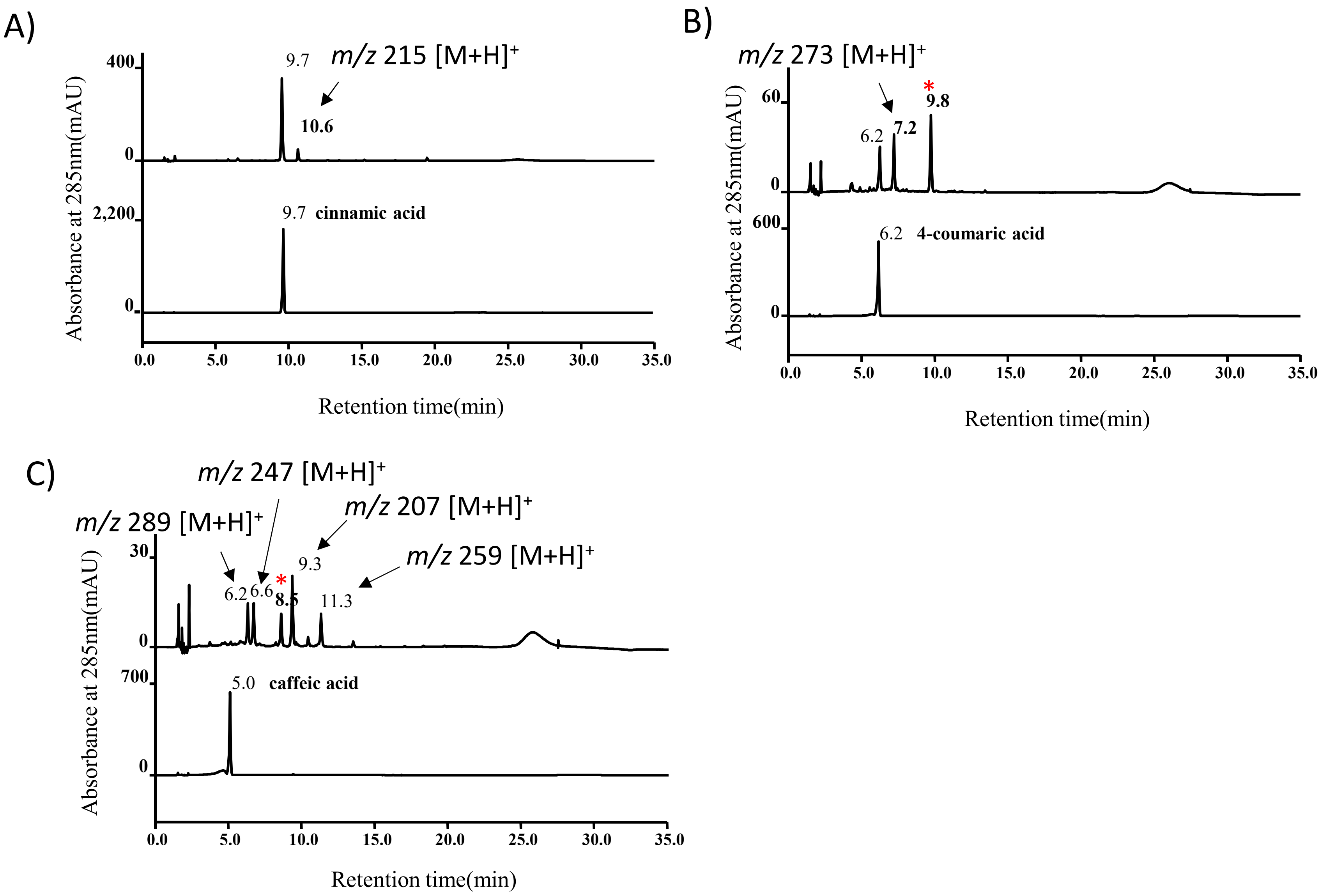

Figure S3. LC/MS/MS analysis of dimer compound.

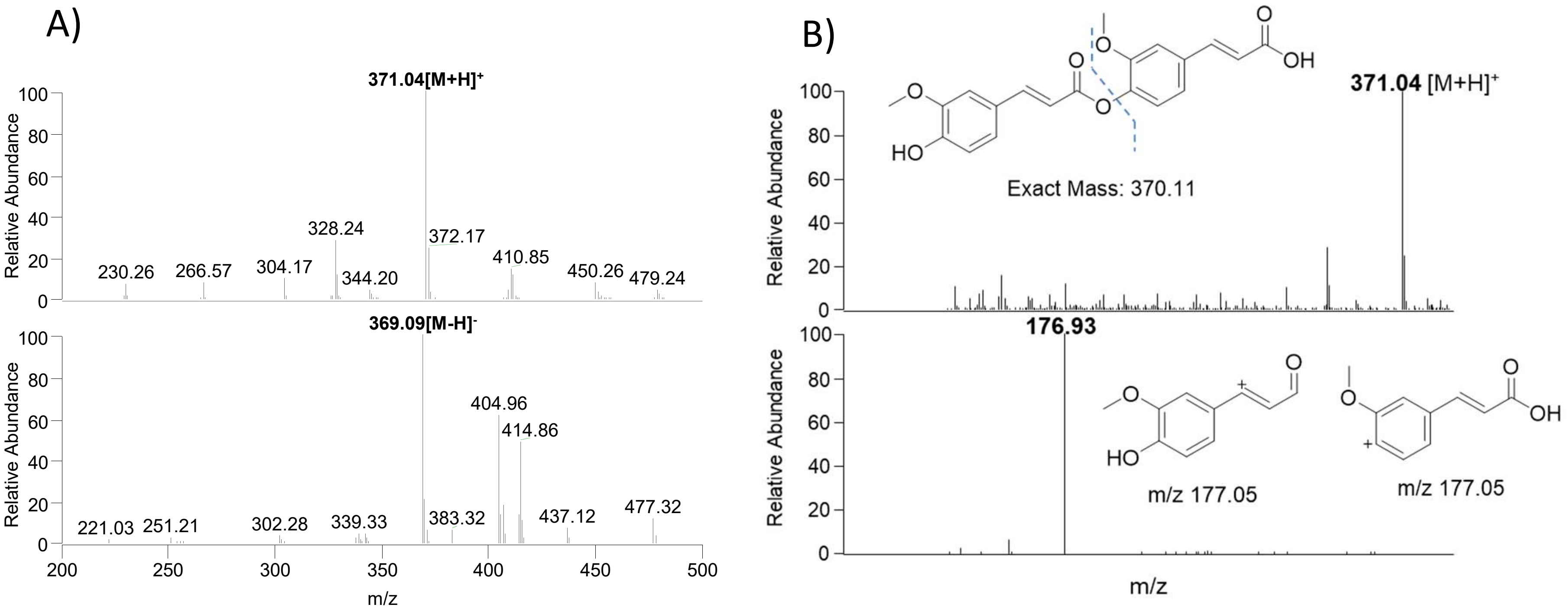

Figure S4. Schematic illustration showing the strategies for constructing the genome engineered strain.

A) Construction of the L-tyrosine overproducing strain of *E. coli* (ΔCOS1)

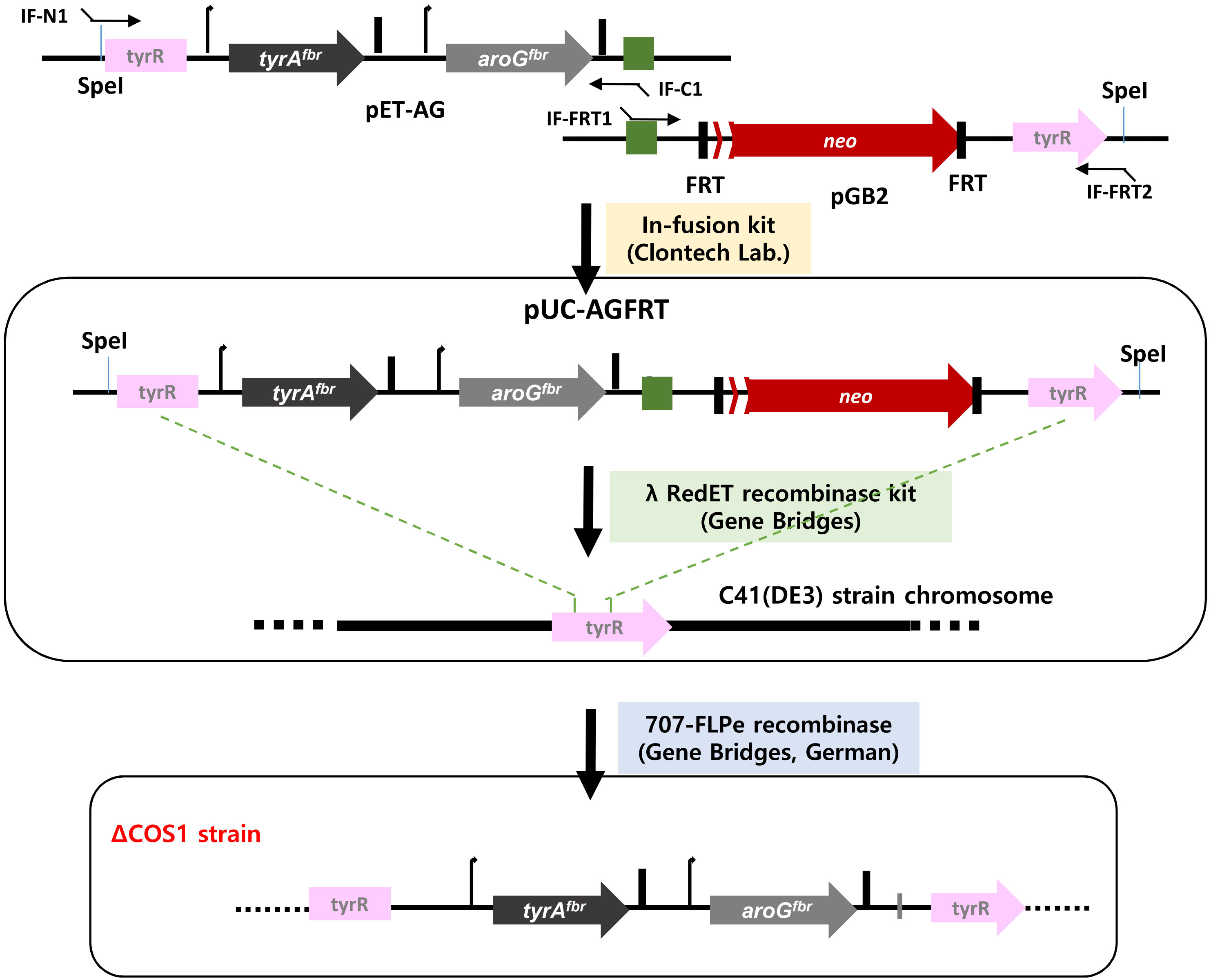

B) Construction of the ferulic acid overproducing strain of *E. coli* (COS6-T5M)

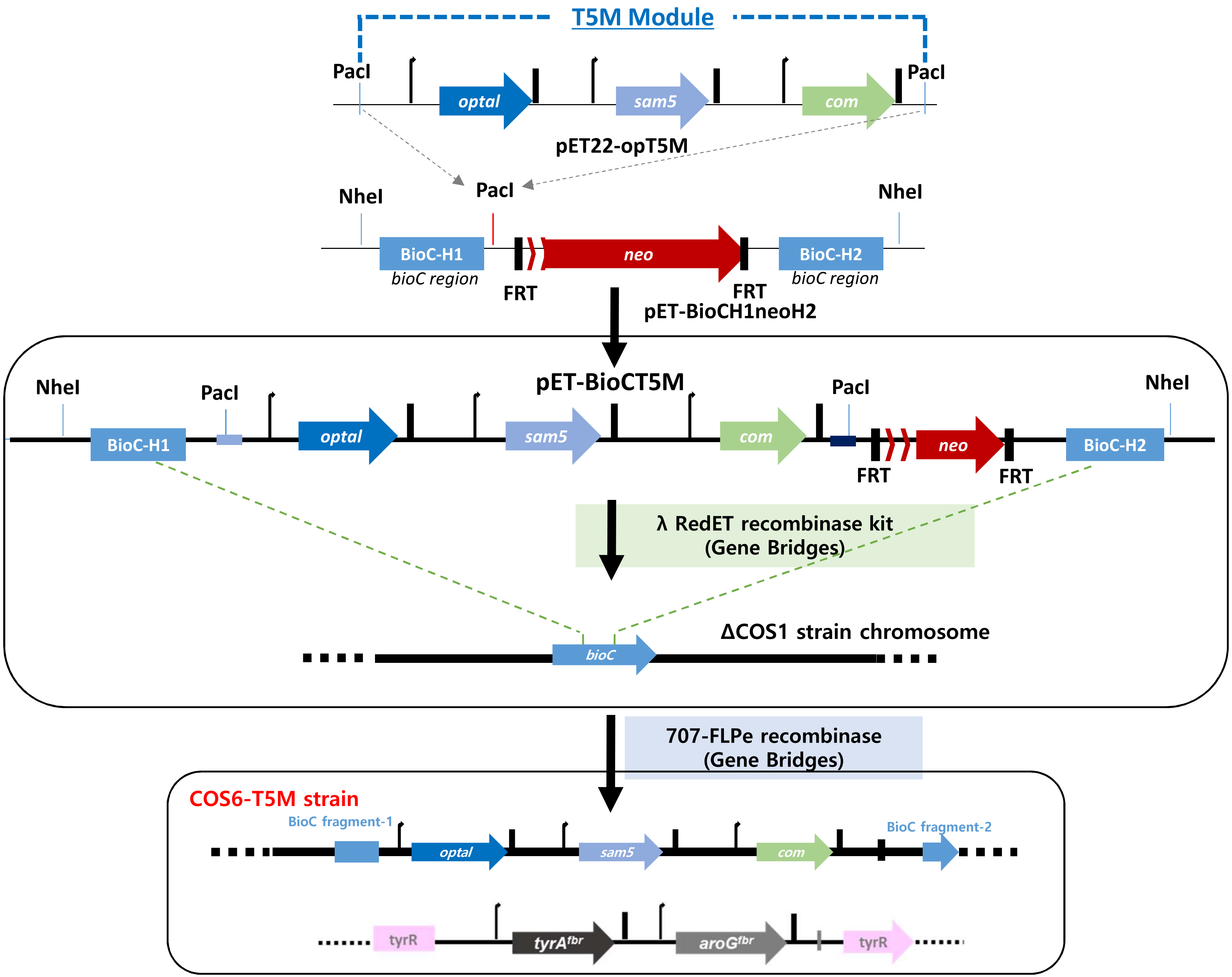

>pnPKS *Piper nigrum* styrylpyrone synthase cDNA

ATGTCGAAGACGGTAGAGGAGATTCGGGCGGCACAGCGGGCGAGGGGACCAGCCGCGGTGCTGGCCATCG
GCACGGCTACCCCGGCCAATGTGGTTTTCCAGGCCGATTATCCGGACTACTACTTTAGGATCACCAAGAGCGA
GCACATGACCGAGCTCAAGGAGAAGTTCCAACGAATGTGTGACAAGTCAATGATAAGGAAGCGGTACATGCA
CTTGTCAGAGGAGCTGCTGAAAAACAACCCTAACATCTGTGCCTACATGGCCCCTTCCCTCGACGCTCGCCAA
GATATGGTGGTGGTGGAGGTACCCAAGCTCGGCAAGGAGGCGGCCGCCAAGGCCATCAAGGAGTGGGGTC
GCCCAAAGTCGGGCATCACCCACCTCATCTTCTGCACTACCTCCGGCGTCGACATGCCCGGCGCCGACTACCA
GCTCACCAAGCTCCTCGGCCTCCGCGCCTCCGTCCGCCGCACCATGATCTATCAGCAGGGCTGCTTCGCCGGT
GGCACTGTCCTCCGCCTTGCCAAGGACCTCGCAGAGAACAATGCGGGCGCGAGGGTCCTCGTCGTCTGCTCC
GAGATCACCGCCGTCACCTTCCGCGGCCCCTCGGAGACTCAACTCGATAACATGGTAGGCCAGGCGCTGTTC
GGCGATGGCGCGGCTGCCATCATTATCGGGGCCGACCCTGACCCTGCCATAGAAAGGCCACTCTTTCAAATG
GTATCTGCAGCTCAGACCATTCTTCCTGACTCGGAGGGAGCCATAGACGGCCATCTCCGAGAAGTGGGTCTAA
CCTTCCACCTCCTCAAGGACGTACCTGGGCTCATCTCAAAGAACATCGAGAAGAGCCTCAAGGAGGAGTTTG
CACCGCTGGGCATCGACGACTGGAACTCGATATTTTGGATAGCTCATCCAGGCGGGCCTGCCATTCTAGACCA
GGTGGAGGCGAAGCTGGGTCTGAAAGAGGACAAGCTGAAGACAACGAGATCAGTTCTGAGAGAGTATGGG
AATATGTCGAGCGCTTGCGTGTTGTTCATACTGGACGAGATGAGGAGGAGGAGCATGGAGGAAGGGAAGAC
GACGACCGGTGAAGGGTTGGATTGGGGAGTTTTGTTTGGTTTTGGGCCGGGTTTGACCGTGGAGACGGTCGT
CTTGCATAGTTTGCCCATCGCCGAGGCCAACTAA
